# DKA with Severe Hypertriglyceridemia and Cerebral Edema in an Adolescent Boy: A Case Study and Review of the Literature

**DOI:** 10.1155/2016/7515721

**Published:** 2016-01-20

**Authors:** Tansit Saengkaew, Taninee Sahakitrungruang, Suttipong Wacharasindhu, Vichit Supornsilchai

**Affiliations:** Division of Endocrinology, Department of Pediatrics, Faculty of Medicine, Chulalongkorn University, Bangkok 10330, Thailand

## Abstract

A 13-year-old adolescent boy with type 1 diabetes mellitus (1b) presented with diabetic ketoacidosis (DKA) and cerebral edema. Grossly lipemic serum and lipemia retinals due to extremely high triglyceride (TG) level were observed without evidence of xanthoma or xanthelasma. Cerebral edema was treated by appropriate ventilation and mannitol administration. Normal saline was carefully given and regular insulin was titrated according to blood sugar levels. Triglyceride levels were reduced from 9,800 mg/dL to normal range within 9 days after conventional treatment was commenced without antilipid medication. Based on our review of the literature, this is the first reported case of confirmed pediatric DKA with severe hypertriglyceridemia and cerebral edema. In patients with DKA and hypertriglyceridemia, clinicians should be mindful of the possibility of associated acute pancreatitis and cerebral edema.

## 1. Introduction

Diabetic ketoacidosis (DKA) is a common presentation of type 1 diabetes mellitus that requires urgent treatment. Diagnostic criteria for DKA include hyperglycemia (BS > 200 mg/dL), ketosis, and metabolic acidosis. The most serious complication in delayed diagnosis of DKA is cerebral edema, a complication that increases patient morbidity and mortality. Acute pancreatitis (AP), peripheral venous thrombosis, pulmonary edema, and rhabdomyolysis are only rarely found in patients with DKA [1].

Severe hypertriglyceridemia (TG > 1,000 mg/dL) is another rare complication found in DKA patients. Severe hypertriglyceridemia can increase risk of acute pancreatitis, especially in patients with TG levels higher than 1,000–1,772 mg/dL [2, 3]. Prevalence of severe hypertriglyceridemia was found in about 8% of adults with DKA [4], but few data have been reported in children with severity ranging from asymptomatic to severe acute pancreatitis. The mechanism of hypertriglyceridemia in DKA that has been proposed involves increased free fatty acid (FFA) secretion from adipocytes by counterregulatory hormone stimulation, which results in decreased clearance of VLDL-TG [5]. Recommended treatment includes intravenous fluid and insulin administration appropriated to DKA treatment guidelines. Plasmapheresis or heparinization was used in some reported cases to decrease triglyceride level in adults [6–13]. The objective of this report is to present and describe the clinical features, laboratory investigations, case management, and natural course of hypertriglyceridemia in a 13-year-old adolescent boy with DKA. A review of the literature relating to hypertriglyceridemia and its manifestations in children with DKA was performed and is included in this report for purposes of comparison with our patient.

We searched all English articles that were published and shown in PubMed, MEDLINE, and Web of Science up to August 2015. The keywords were DKA, hypertriglyceridemia, cerebral edema, and acute pancreatitis. We selected only publications (retrospective cohort studies and case reports) about children, adolescents, and young adults who presented with DKA and hypertriglyceridemia. All clinical and laboratory data including age at presentation, initial TG level, diagnosis and treatment of acute pancreatitis, and duration of TG return to normal range were reviewed and summarized.

## 2. Case Presentation

**Figure 1:**
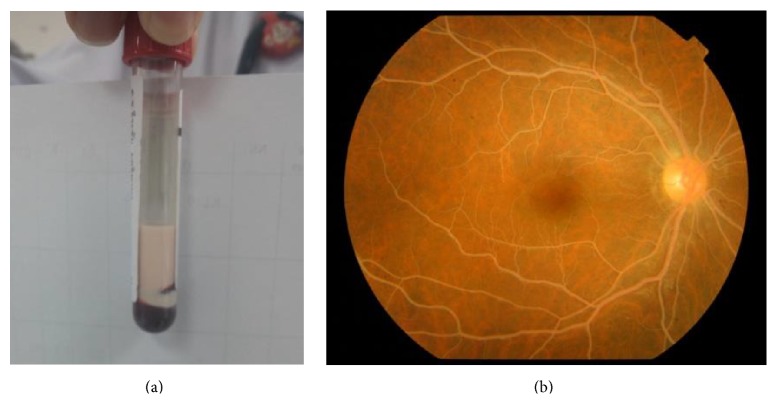
Images describing lipemic serum (a) and lipemia retinals, whitish, creamy vessels of retina (b).

A 13-year-old boy with type 1 diabetes mellitus (T1DM) presented at the Emergency Department of King Chulalongkorn Memorial Hospital (Bangkok, Thailand) with Kussmaul breathing and confusion, after having malaise and polyuria for one month and epigastric pain for 2 days. He was diagnosed with T1DM at another hospital one year earlier and was being treated with insulin, metformin, and glipizide. Our patient reported having discontinued all medications for 6 months prior to this admission. His maternal grandmother was diagnosed with type 2 diabetes mellitus at the age of 40 and her condition was reported as being effectively controlled with oral hypoglycemic drugs. No family history of dyslipidemia was reported. On admission, our patient was confused, agitated, and disoriented regarding time, place, and persons. Vital signs revealed body temperature of 38.5°C, pulse rate 128, respiratory rate 26/min with Kussmaul pattern, and blood pressure 156/102 mmHg. Anthropometric measurements showed weight of 43 kg, height 152 cm, and body mass index (BMI) 18.61 kg/m^2^. There was no eruptive or tuberous xanthoma or xanthelasma at the skin. Ophthalmologic examination revealed presence of whitish, creamy vessels of retinas, called lipemia retinals (Figure 1(a)). No focal neurologic deficit was noted.

**Figure 2:**
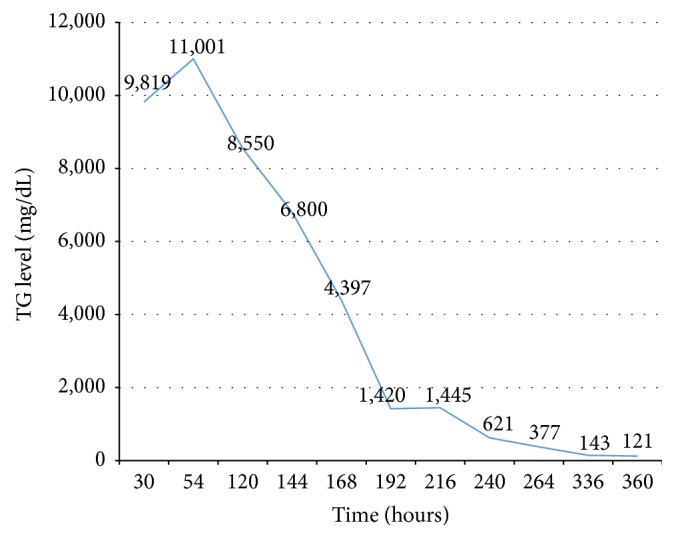
Triglyceride levels after DKA treatment.

At presentation, our patient was found to have DKA, with initial BS of 513 mg/dL, venous blood gas with pH 7.062, pCO_2_ 22.4 mmHg, HCO_3_ 6.4, and BE −21.7 mEq/L, and serum ketone 3.2 mmol/L. His blood demonstrated a grossly lipemic appearance (Figure 1(b)) and his lipemic condition disturbed the results of other biochemical blood investigations. Cerebral edema was diagnosed according to 1 major criterion (alteration and fluctuation of consciousness) and 2 minor criteria (lethargy and diastolic blood pressure >90 mmHg). CT of the brain was not performed due to the unstable clinical condition of our patient. He was intubated due to deterioration of consciousness and cerebral edema. Mannitol (0.5 mg/kg) was subsequently administered and 3% NaCl was given intravenously to maintain a hypernatremic state for purposes of decreasing intracranial pressure. Normal saline was carefully given and regular insulin was then started and titrated to 0.2 units/kg/hr according to patient blood sugar levels. He was out of DKA as defined by pH > 7.3, bicarbonate > 15 mmol/L, and serum ketone negative, within 24 hours. Lipid profiles were analyzed on the second day of admission, with results revealing triglyceride of 9,810 mg/dL, cholesterol 705 mg/dL, LDL 254 mg/dL, and HDL 6 mg/dL. Patient had no abdominal pain and serum amylase was 57 IU/L. Therefore, acute pancreatitis was excluded. Triglyceride level was reduced to 377 mg/dL within 9 days of admission (Figure 2) without the use of antilipid medication. Our patient was extubated within 2 days of admission after recovery from DKA and cerebral edema. His father's lipid profiles showed cholesterol of 215 mg/dL, triglyceride 111 mg/dL, LDL 144 mg/dL, and HDL 50 mg/dL. His mother's lipid profiles showed cholesterol of 167 mg/dL, triglyceride 75 mg/dL, LDL 101 mg/dL, and HDL 47 mg/dL.

After discharge from the hospital, T1DM treatment with basal-bolus insulin regimen was commenced. Our patient continued to have HbA1c-related complications (10–15%) due to poor self-treatment compliance. Although he developed DKA twice as a result, his TG levels remained within normal range.

## 3. Discussion

Two large series reported data in adult DKA with severe hypertriglyceridemia [4, 19]. Fulop and Eder found that 15 of 136 (11%) DKA patients had severe hypertriglyceridemia (TG > 1,000 mg/dL), but only one patient developed acute pancreatitis [19]. Incidence of severe hypertriglyceridemia was similarly found in 8 of 100 (12.5%) adult DKA patients in the other of the two studies, with half of the patients developing acute pancreatitis [4]. In children, most cases were individually presented in patient case reports (Table 1). All reported cases had TG levels higher than 1,000 mg/dL, supporting the hypothesis that elevated TG (>1,000 mg/dL) is a risk factor for acute pancreatitis [2, 3]. However, 3 out of 11 patients (2.8%) did not develop acute pancreatitis. One pediatric patient did not develop acute pancreatitis, even though he had extremely high TG level (14,461 mg/dL) [6]. As such, other risk factors for this condition need to be identified. Accordingly, clinical investigations for acute pancreatitis should be routine in all cases presenting with TG level higher than 1,000 mg/dL to ensure early and proper management.

The postulated mechanism of hypertriglyceridemia in DKA patients centers on lack of insulin action, which activates lipolysis in adipose tissue that then results in free fatty acid (FFA) formation and increases in VLDL formation by the liver [19]. In addition, insulin normally inhibits ApoC-III expression, which plays a major role in inhibiting lipoprotein lipase (LPL) and hepatic lipase (HL). This results in decreased hydrolysis and delayed VLDL-TG clearances from plasma. As a result, ApoC-III increases in an insulin-deficient state, with a subsequent increase in plasma TG level [5]. Severe hypertriglyceridemia-induced pancreatitis develops as a result of increased FFA levels that are produced from triglyceride hydrolysis by pancreatic lipase and insulin deficiency. High FFA levels cause acute pancreatitis as a result of capillary endothelium and pancreatic acinar cell destruction [14, 20].

Appropriate management of DKA with hypertriglyceridemia includes intravenous fluid and insulin administration according to DKA guideline, because the major mechanism of hypertriglyceridemia is insulin deficiency. Plasma TG level was gradually reduced to less than 500 mg/dL within 3–17 days in most cases. However, 9 of 15 patients reviewed by Fulop and Eder had normal plasma TG concentrations for up to 6 months without any lipid-lowering agent [19]. One patient reported by Lutfi et al. who had extremely high TG level (16,334 mg/dL) with renal insufficiency and who failed with conservative treatment was successfully treated by plasmapheresis [11]. TG levels were reduced from 5,093 to 1,157 mg/dL in that study. Heparinization was also reported as an alternative treatment for hypertriglyceridemia in adult DKA patients, together with fluid and insulin administration [8]. In that study, TG levels were reduced from 8,701 mg/dL to normal range within 2 days of treatment without abnormal bleeding.

Cerebral edema was suspected in 3 pediatric cases from the literature review and one case from the present report. All 3 cases from the literature had abnormal pupillary reflex with unconsciousness, but all had spontaneous recovery without treatment by mannitol or hypertonic saline. To our knowledge and based on our review of the literature, the association between severe hypertriglyceridemia and cerebral edema is unclear. The hypothesized mechanism is that hyperviscosity due to extremely high TG level likely causes decreased cerebral blood flow, which may correlate with cerebral edema [21]. Yuen et al. found that severe dehydration and hypertriglyceridemia aggravated cerebral hypoperfusion, which had the effect of worsening cerebral edema.

The weakness of our case was that serum lipase test was not done and contrast enhanced CT of the abdomen was not performed. Even though the patient had no abdominal pain and normal amylase level, acute pancreatitis could not be absolutely excluded.

In this case report, we presented a pediatric DKA patient with cerebral edema and severe hypertriglyceridemia. In patients with DKA and hypertriglyceridemia caused by severe insulin deficiency, clinicians should be mindful of the possibility of associated acute pancreatitis and cerebral edema. Triglyceride levels were gradually decreased to normal range within 9 days after standard intravenous fluid and insulin administration was commenced.

## Figures and Tables

**Table 1:** Literature review of pediatric DKA patients with hypertriglyceridemia.

Patients	Age (year)	Peak TG (mg/dL)	Acute pancreatitis	Cerebral edema	Treatment with antilipid medication	Time to normalized TG level (days, (TG level))
Cywinski et al., 1965 [14]	12.5	>1,000	Yes	Suspected	No	7 (232)
Blackett et al., 1986 [6]	13	14,461	No	Suspected	No	7 (122)
Slyper et al., 1994 [15]	14	3,119	Yes	Suspected	No	NA
Kadota-Shinozaki et al., 1997 [16]	19	3,386	Yes	No	No	2 (483)
Hahn et al., 2010 [10]	20	15,000	Yes	No	No	3 (506)
Williamson et al., 2012 [12]	8	10,852	No	No	No	17 (NA)
Lutfi et al., 2012 [11]	10	16,334	Yes	No	Fenofibrate, plasmapheresis	1.5 (1,100)
Kota et al., 2012 [17]	12	1,020	Yes	No	No	3 (340)
Aboulhosn and Arnason, 2013 [13]	18	1,724	Yes	No	No	NA
Wolfgram and Macdonald, 2013 [18]	10	8,300	Yes	No	No	NA

Our case	**13**	**9,810**	**No**	**Yes**	**No**	**9 (377)**

NA, not available.
